# A PHD-zinc finger-mediated mechanism for PHF21B recognition of H3K36me3

**DOI:** 10.61373/gp026l.0042

**Published:** 2026-06-09

**Authors:** Q. Ma, J. Licinio, M.L. Wong

**Affiliations:** 1 Department of Psychiatry and Behavioral Sciences, College of Medicine, State University of New York, Upstate Medical University, Syracuse, NY 13210, USA; 2 Department of Neuroscience & Physiology, College of Medicine, State University of New York, Upstate Medical University, Syracuse, NY 13210, USA

## Abstract

HF21B is an epigenetic reader that modulates synaptic plasticity-related genes and social memory (1). Although PHF21B is known to bind H3K36me3, the domain responsible for this interaction has remained uncharacterized. Here, we used co-immunoprecipitation with PHF21B deletion mutants in HEK293T cells to show that the PHD-zinc finger domain is essential for H3K36me3 recognition, whereas the C-terminal coiled-coil region is dispensable. These findings identify the PHD-zinc finger domain as the critical H3K36me3-binding module of PHF21B, with implications for neurological disorders linked to chromosome 22q13.3 deletion, including Phelan-McDermid syndrome (2, 3).

## Introduction

H3K36me3 is a histone modification deposited co-transcriptionally at actively transcribed gene bodies, where it regulates RNA polymerase II elongation, alternative splicing, and chromatin architecture (4). This mark is recognized by a variety of reader domains, among which plant homeodomain (PHD) zinc fingers constitute a functionally versatile class of effectors capable of binding methylated histone H3 tails and recruiting transcriptional regulatory complexes to chromatin (5, 6). Disruption of H3K36 methyltransferases such as *SETD2*, *NSD1*, *NSD2*, and *SETD5* causes syndromic neurodevelopmental disorders with intellectual disability, autism spectrum features, and seizures (4, 7), highlighting the sensitivity of brain development to altered H3K36 methylation dosage.

**Figure 1.**
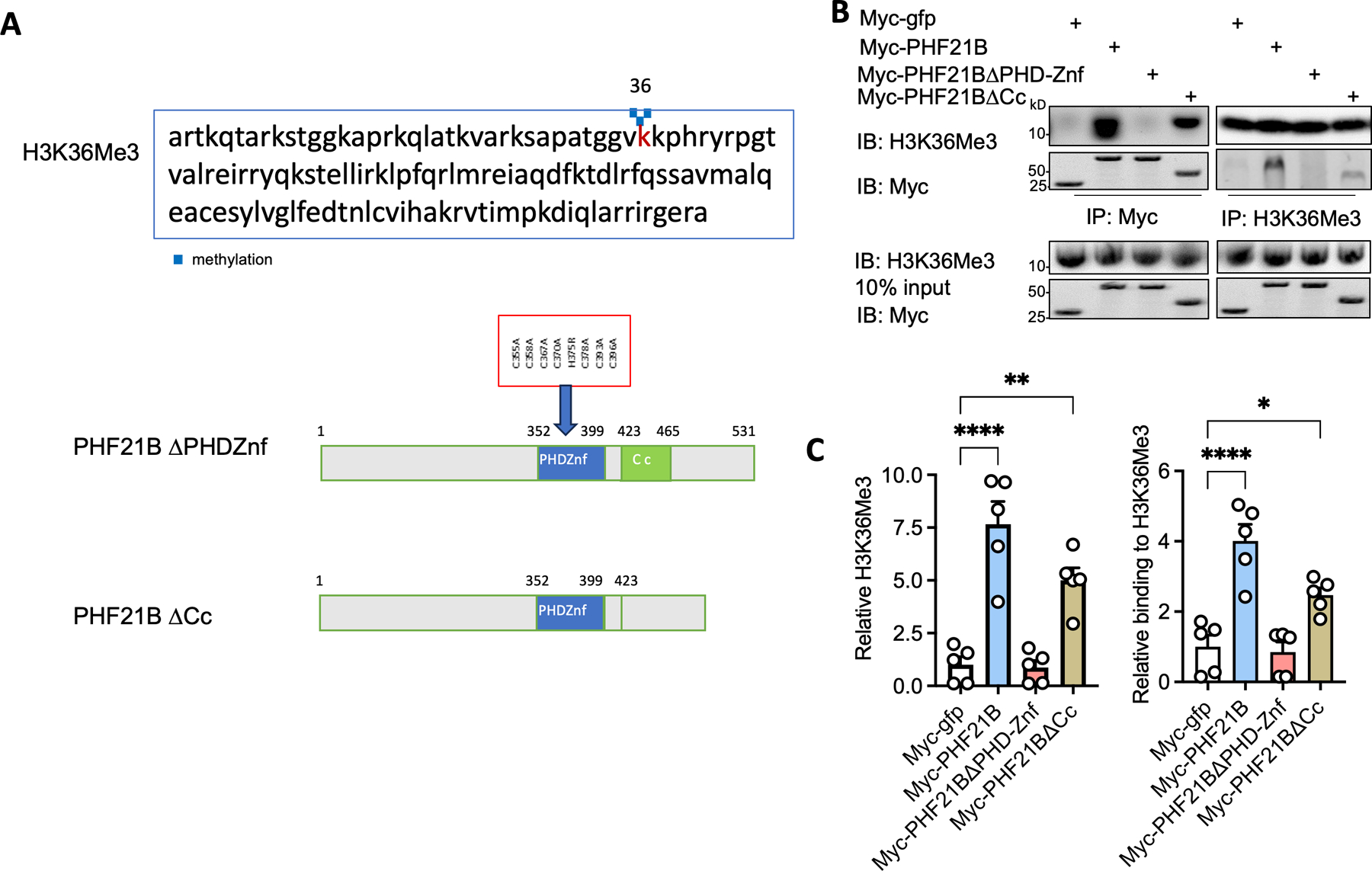
The PHD-zinc finger domain of PHF21B is essential for H3K36me3 recognition. (A) Schematic of H3K36me3-modified H3 tail, PHF21B C355A/C358A/C367A/C370A/H375R/C378A/C393A/C396A in the PHD-Znf domain (ΔPHD-Znf, blue) or the C-terminal coiled-coil region (ΔCc, green). Numbers and letters in the red rectangle indicate amino acid substitutions. (B) Co-immunoprecipitation of Myc-tagged PHF21B constructs with H3K36me3 in HEK293T cells, followed by immunoblotting (IB) for H3K36me3 and Myc. Input and reciprocal immunoprecipitations (IP: Myc; IP: H3K36me3) show that full-length PHF21B strongly associates with H3K36me3, ΔCc shows reduced binding, and ΔPHD-Znf shows no binding. (C) Quantification of relative H3K36me3 levels (left) and PHF21B construct binding to H3K36me3 (right) from five independent experiments. Data represent mean ± SEM; ∗ *P* < 0.05; ∗∗ *P* < 0.01; ∗∗∗∗ *P* < 0.0001; one-way ANOVA with Sidak’s post hoc test. For each experiment, co-immunoprecipitated H3K36me3 and PHF21B signals were quantified by densitometry of immunoblot bands, normalized to the corresponding input signal, and expressed relative to the wild-type PHF21B condition. Group means were compared by one-way ANOVA with Sidak’s post hoc test for multiple comparisons; *n* = 5 independent experiments per condition.

To determine which domain of PHF21B mediates H3K36me3 recognition, we generated two mutants: ΔPHD-Znf (PHF21B C355A/C358A/C367A/C370A/H375R/C378A/C393A/C396A in residues 352–399, which encompass the PHD-zinc finger domain) and deletion mutant ΔCc (lacking residues 423–465, the C-terminal coiled-coil region; Figure 1A). The boundaries of each mutant were chosen to encompass the predicted extent of a single structural module, the PHD-zinc finger or the coiled-coil region, so that each construct removes one candidate H3K36me3-interacting module while leaving the remainder of PHF21B intact, allowing the contribution of each domain to be assessed independently. Myc-tagged constructs were expressed in HEK293T cells and subjected to co-immunoprecipitation. For further information on Material and Methods, see Online Supplementary Material. Wild-type PHF21B and PHF21B ΔCc both pulled down H3K36me3, whereas PHF21B ΔPHD-Znf showed no detectable interaction (Figure 1B, left panels). Reciprocal co-immunoprecipitation using anti-H3K36me3 antibodies confirmed these results: wild-type PHF21B and ΔCc were recovered, but PHF21B ΔPHD-Znf was absent (Figure 1B, right In conclusion, we identify the PHD-zinc panels). Quantification across five independent experiments demonstrated that full-length PHF21B exhibited the highest H3K36me3 binding, ΔCc showed intermediate binding (Figure 1C; *P* < 0.05 and *P* < 0.01), and ΔPHD-Znf showed no binding (Figure 1C; *P* < 0.0001; one-way ANOVAs with Sidak’s post hoc tests). These results establish that the PHD-zinc finger domain is necessary for PHF21B recognition of H3K36me3; the C-terminal coiled-coil region, by contrast, plays a modulatory but dispensable role. The intermediate binding retained by the ΔCc mutant indicates that, although the coiled-coil region is not required for the interaction, it contributes to full-affinity H3K36me3 engagement. This partial reduction suggests that the coiled-coil region acts in a supportive capacity, for example, by stabilizing the conformation of the adjacent PHD-zinc finger or by promoting additional contacts with the nucleosomal context, rather than directly contacting the H3K36me3 mark itself.

Our findings are consistent with the known capacity of PHD fingers to function as histone methyllysine readers, a role originally described for H3K4me3 recognition but now extended to H3K36me3 (5, 6). Structural analyses of other PHD-zinc finger-containing proteins, including ZMYND11, have shown that tandem reader modules cooperate in H3K36me3 recognition, with the PHD finger contributing to chromatin association alongside PWWP and bromodomains (8). By analogy, the partial reduction in H3K36me3 binding observed for the ΔCc mutant suggests that the coiled-coil region of PHF21B contributes to chromatin engagement in a supportive, accessory capacity, consistent with a multi-module reader in which the PHD-zinc finger provides the essential H3K36me3 contact and adjacent regions tune the overall affinity. The preferential binding of PHF21B to H3K36me3 over H3K4me3 (1), combined with the present domain mapping, suggests a mechanistic specialization in which the PHD-zinc finger of PHF21B is tuned for recognition of actively transcribed gene bodies rather than promoter-associated marks. This gene-body specialization provides a direct mechanistic link to the function of PHF21B in neurons: because H3K36me3 marks the bodies of actively transcribed genes, recognition of this mark by the PHD-zinc finger positions PHF21B to act selectively at synaptic plasticity genes during their transcription. PHF21B depletion reduces the expression of these synaptic plasticity genes and impairs social memory in mice (1); the present domain mapping indicates that an intact PHD-zinc finger–H3K36me3 interaction is the molecular event required for PHF21B to engage these gene bodies, and therefore that loss of this interaction likely underlies key aspects of the role of PHF21B in the transcriptional regulation of neuronal genes.

In conclusion, we identify the PHD-zinc finger domain as the essential structural determinant for PHF21B recognition of H3K36me3. By defining the PHD-zinc finger–H3K36me3 interface as the molecular interaction that enables PHF21B to read active chromatin, these data identify a specific, well-defined interface that could be targeted to modulate PHF21B activity. This is of particular relevance to Phelan-McDermid syndrome and related disorders associated with 22q13.3 deletion, in which reduced PHF21B dosage is expected to diminish recognition of H3K36me3 at synaptic plasticity gene bodies and thereby contribute to the dysregulation of synaptic plasticity genes and social memory observed upon PHF21B loss (1–3). Defining this interface, therefore, provides a rational starting point for future strategies to restore or compensate for PHF21B reader function, although implementing such interventions will require further structural and functional investigation. Future structural studies of the PHF21B PHD-zinc finger in complex with H3K36me3 peptides will provide atomic-level insight into this recognition mechanism and enable structure-guided therapeutic design.

## Supplementary Material

Supplementary Material
