# Supplementary material for: A PHD-zinc finger-mediated mechanism for PHF21B recognition of H3K36me3
Source: Genom Psychiatry. Author manuscript; Available in PMC 2026 Jun 27. (PMC13309230; doi:10.61373/gp026l.0042)
Supplement: Supplementary Material [file NIHMS2187564-supplement-Supplementary_Material.pdf]

Online Supplementary Material

A PHD-zinc finger-mediated mechanism for PHF21B recognition of H3K36me3

Qi Ma, Julio Licinio*, Ma-Li Wong*
***Corresponding authors' email:**
LicinioJ@upstate.edu (J.L.); wongma@upstate.edu (M.-L.W.)
***Genomic Psychiatry* 2026 ▪ https://doi.org/10.61373/gp026l.0042**

Supporting Online Material

Materials and Methods

Plasmid construction. PHF21B deletion mutants were generated using standard molecular cloning techniques. PHF21B ΔPHD-Znf has several point mutations in the PHDZnf domain (residues 352–465), while PHF21B ΔCc lacks residues 423–531. All constructs were Myc-tagged for detection.

Cell culture and transfection. HEK293T cells were cultured in DMEM supplemented with 10% FBS. Transfections were performed using Lipofectamine 3000 (Thermo Fisher Scientific) according to the manufacturer's instructions.

Co-immunoprecipitation and western blotting. Cells were lysed 48 h post-transfection in RIPA buffer without SDS. Immunoprecipitation was performed using anti-Myc antibodies (Cell Signaling Technology) or anti-H3K36me3 antibodies (Thermo Fisher Scientific) with protein A/G agarose beads. Precipitated complexes were resolved by SDS-PAGE and immunoblotted using standard protocols. Band intensities were quantified with ImageJ and normalized to input.

Statistical analysis. Data are presented as mean ± SEM from three independent experiments. Statistical comparisons were performed by one-way ANOVA with Sidak's post hoc test. Significance thresholds: $^{**}P < 0.01$; $^{***}P < 0.001$; $^{****}P < 0.0001$.
